# The Immune Response to Nematode Infection

**DOI:** 10.3390/ijms24032283

**Published:** 2023-01-23

**Authors:** Michael Stear, Sarah Preston, David Piedrafita, Katarzyna Donskow-Łysoniewska

**Affiliations:** 1 Department of Animal, Plant and Soil Sciences, AgriBio, La Trobe University, Bundoora, VIC 3086, Australia; 2 School of Science, Psychology and Sport, Federation University Australia, Mt Helen, VIC 3350, Australia; 3 Department of Experimental Immunotherapy, Faculty of Medicine, Lazarski University, ul. Świeradowska 43, 02-662 Warsaw, Poland

**Keywords:** nematode, immunity, TH2, IgA, eosinophil, IgE, mast cell, regulatory T cell, immunomodulation, major histocompatibility complex

## Abstract

Nematode infection is a major threat to the health of humans, domestic animals and wildlife. Nematodes vary in their effect on the host and in the mechanisms underlying immunity but the general features are becoming clear. There is considerable variation among individuals in resistance to infection and much of this variation is due to genetic variation in the immune response. The major histocompatibility complex has a strong influence on resistance to infection but other genes are collectively more important. Resistant individuals produce more IgA, eosinophils, IgE and mast cells than susceptible individuals and this is a consequence of stronger type 2 (Th2) immune responses. A variety of factors promote Th2 responses including genetic background, diet, molecules produced by the parasite and the location of the infection. A variety of cells and molecules including proteins, glycolipids and RNA act in concert to promote responses and to regulate the response. Nematodes themselves also modulate the host response and over 20 parasite-derived immunomodulatory molecules have been identified. Different species of nematodes modulate the immune response in different ways and probably use multiple molecules. The reasons for this are unclear and the interactions among immunomodulators have still to be investigated.

## 1. Introduction

Nematodes cause disease and death in humans, domestic animals including livestock, wild animals and plants. Over a quarter of the human population is affected [1] while nematode infection costs livestock producers hundreds of millions of dollars every year [2,3,4]. In addition, nematode infections can influence the size of wild populations [5].

Even subclinical infections can cause immense harm. Nematode infection impairs cognitive development in humans. Even light infections can reduce growth rates and productivity in livestock [6,7]. Feral sheep with heavier nematode infections are more likely to die when food is scarce [8]. The threat posed by nematodes to human and animal health is worsening as parasites evolve resistance against the drugs used to treat them while global warming is allowing pathogenic nematodes to expand their host range [9]. More sustainable methods of nematode control are urgently needed [10].

The development of sustainable methods of nematode control requires a good understanding of the interaction between hosts and their parasites, particularly the immune response. The immune response to metazoan parasites including nematodes is complex. Nematodes produce thousands of molecules and hundreds of these are recognised by the immune response [11]. One consequence is that, unlike many viral infections, nematode molecules are not evolving rapidly to evade the antibody response [12]. If protection evolves the recognition of many molecules, then the selective pressure from the immune system on a single gene product is quite low. Even if a molecule mutates and evades the immune response against it, the parasite may still be killed by the immune response against other molecules. Immunity usually requires the recognition of multiple molecules, which may help explain why producing vaccines has been so difficult. However, some molecules may be more important for immunity than others. A carbohydrate larval antigen (CARLA) from *Trichostrongylus colubriformis* appears particularly important for immunity and the IgA response to this glycolipid has shown promise as a target for diagnostic testing [13,14].

The immune response is responsible for resistance to infection but can also cause immune-mediated diseases such as multiple sclerosis, diabetes, asthma and inflammatory bowel disease. The immune response is compartmentalised [15]; the Th2 response underlies resistance to gastrointestinal nematodes [16] and can be divided into three overlapping phases: induction, expression of protective mechanisms and regulation. The parasite in turn attempts to suppress or evade the immune response; a process known as immunomodulation. The four components (induction, expression of protective mechanisms, regulation and immunomodulation) will be considered sequentially although there is considerable overlap in their timing.

## 2. Induction of the Th2 Response

The Th2 response is sometimes labelled ‘anti-inflammatory’ in comparison with the Th1 response but this can be misleading. Acute inflammation is driven by neutrophils [17] which are activated by the Th1 response but chronic inflammation is driven by mast cell degranulation [17] and mast cell development and recruitment is driven by the Th2 response. Similarly, the Th2 response is sometimes labelled as the antibody promoting response in contrast to the Th1 response which promotes cell-mediated immunity. Again, this can be misleading. Th2 responses promote eosinophil and mast cell responses [17].

The mechanisms that generate the Th2 immune response are reasonably well understood, at least in mice and humans, although some of the details are still unclear. Prior to nematode infection, thymocytes develop and are selected in the thymus producing a variety of naïve T cells that are capable of recognising and responding to foreign molecules presented on the surface of antigen presenting cells (APC). The three major types of APC are dendritic cells (DC), macrophages and B cells. These naïve T cells leave the thymus [17].

Naïve B cells are generated in the bone marrow in mice and humans but in sheep, cattle, and presumably related species of the bovidae, they undergo further maturation in the Ileal Peyer’s patches [18]. Together with naïve T cells, the naive B cells circulate through the blood and lymph passing through lymph nodes, Peyer’s Patches and isolated lymphoid follicles. Naïve B and T cells continue to recirculate around the body until they recognise fragments of processed parasite molecules on dendritic cells.

Following infection with gastrointestinal nematodes, dendritic cells ingest and process parasite molecules and migrate to the draining lymph node or to unencapsulated lymphoid tissue [17]. Processed parasite molecules are presented on the surface of dendritic cells by Major Histocompatibility Complex (MHC) class II molecules. MHC molecules play a crucial role in antigen presentation. A particular MHC allele in the DRB1 locus of sheep has been associated with resistance to both deliberate [19] and natural, predominantly *Teladorsagia circumcincta*, infection [20,21,22]. The same allele DRB1*1101 has been associated with resistance, as shown by fewer eggs in the faeces or reduced numbers of worms [23,24,25,26], in three breeds of sheep (Scottish Blackface, Suffolk and Texel). This is one of the clearest and most convincing associations of the MHC with parasitic infection but associations with nematodes have also been reported in mice [27] and cattle [28]. However, the resistant allele does not preferentially recognise a single important parasite molecule; it complements other alleles in heterozygotes so that together both MHC molecules allow the host immune system to recognise a better, and presumably larger, set of parasite molecules [23].

The classical procedure used by quantitative geneticists to indicate the importance of a polymorphism is to describe the proportion of the total genetic variance accounted for by that SNP or allele. However, estimates of genetic variance are imprecise unless extremely large numbers of individuals are studied. The DRB1*1101 allele accounts for 10–30% of the genetic variance in faecal egg counts when the samples are collected on one day [21,23,24]. This means that the MHC is the most important single gene or system influencing resistance to nematode infection in sheep and presumably other species as well. However, the genes outside the MHC taken together have a greater influence than the MHC. In other words, the effect of the MHC is strong but not overwhelming.

Naïve lymphocytes that recognise processed antigen on the surface of dendritic cells are activated. T cells are activated in part by dendritic cells while B cells are activated by dendritic cells and activated T cells. Activated T cells develop into a variety of specialised T cells including helper T cells (Th) or regulatory T cells (Treg) [17]. Th initiate and elaborate immune responses while Treg prevent inappropriate immune responses against self-molecules but also suppress the established immune response.

Activated B cells move within the lymph node or unencapsulated lymphoid tissue and form germinal centres where they undergo class switching and somatic mutation to produce high affinity antibodies against the specific parasite molecule that they have recognised. The activated cells leave the lymphoid tissue and migrate into the tissues, a process driven by the expression and binding of specific molecules. In the gastrointestinal tract, activated cells accumulate in the lamina propria underlying the mucosal surface.

The activation process differs for different types of specialised T cell. At least three signals drive the development of naïve T cells into specialised T helper cells: The T-cell receptor (TCR) must bind to the processed parasite molecule which is presented by the class II MHC molecule; additional molecules on T cells interact with molecules on dendritic cells and provide additional signals while cytokines also bind receptors on T cells.

Dendritic cells consist of several subpopulations. The subpopulation that assists differentiation into Th2 cells following nematode infection expresses CD301b and PDL2 [29]. This subpopulation also expresses several molecules that drive Th2 development, including the costimulatory molecule OX40L which binds to OX40 on the T cell, and jagged 1 which binds to the Notch molecule as well as the IL-33 receptor [29,30]. Several transcription factors are active in these DC and assist Th2 differentiation including interferon regulatory factor 4 (IRF4) [31,32] and Krüppel-like factor 4 (KLF4) [33]. IRF4 induces expression of IL-10 and IL-33 [31]. IRF4 positive DC can also promote other responses including Th17-mediated responses. KLF4 is required for the promotion of Th2 responses but does not contribute to the Th17 response [30,33].

For Th2 development, the most important cytokine is interleukin 4 (IL-4). Several immune cells including mast cells [34] produce interleukin 4 (IL-4) and leukotrienes [34] which stimulate group 2 innate lymphoid cells (ILC2). These cells are a major source of IL-4 which drives Th2 cell activation and expansion [35]. IL-4 activates signal transducer and activator of transcription 6 (STAT6) which upregulates the transcription factor GATA3 which in turn collaborates with IL-2 induced STAT5 to upregulate the genes needed in Th2 differentiation. The key cytokines produced by Th2 cells (type 2 cytokines) are IL-4, IL-5, IL-9 and IL-13 [30].

Weak stimulation of the TCR and relatively low concentrations of antigen trigger Th2 responses rather than Th1 responses [36]. The strength of the TCR signal depends upon the number of peptide MHC complexes on the surface of the APC, the affinity of the binding and upon the number of costimulatory molecules on the APC surface [30]. T cells receiving strong signals increase the expression of IL-12Rβ2. IL-12 binding promotes the development of Th1 rather than Th2. In contrast, weak signalling induces GATA3 [30].

A number of factors preferentially promote the development of a Th2 response including genetic background, diet, the immunogenic molecules produced by the parasite and the location of the infection; infections of mucosal surfaces such as the lung and the gastrointestinal tract favour Th2 responses [17]. However, the relative importance of these factors and their interactions are not fully understood.

Genetic background seems especially important. For example, there is considerable variation among inbred strains of mice in response to the intestinal nematode *Heligmosomoides polygyrus*. Balb/c mice expel the parasite within 6–8 weeks while CBA and C3H mice take longer than 20 weeks [37]. Quantitative genetic analysis in sheep and cattle has consistently shown heritabilities of a single faecal egg count following natural or deliberate infection to be between 0.1 and 0.4 [28,38]. These heritabilities are similar to the heritability of milk production in dairy cattle or growth rate in beef cattle and high enough to justify selective breeding of sheep for resistance to nematode infection [39]. Faecal egg count is a convenient but relatively imprecise measure of resistance to nematode infection. Other measures such as worm length [40], eosinophil response [41] or nematode-specific IgA activity [42] have even higher heritabilities.

Parasite molecules can promote Th2 responses. Complex glycans can bind C type lectin receptors and trigger Th2 responses [43]. One example is the glycoprotein omega-1 which is secreted by the eggs of *Schistosoma mansoni*. This molecule is a T2 ribonuclease. It binds to the mannose receptor on dendritic cells and following internalisation it degrades both ribosomal and messenger RNA to impair protein synthesis [44]. These dendritic cells preferentially prime naïve T cells to become Th2 cells. Allergen extracts from the house dust mite *Dermatophagoides farina* bind to dectin-2 on murine DC and trigger type 2 responses, partly through the release of cysteinyl leukotrienes [43].

Diet can influence the immune response. Short chain fatty acids preferentially induce the production of DC in the bone marrow that express reduced amounts of OX40, PDL2 and CD86. These DC are less able to prime Th2 responses in the lung [45]. In ruminants, infection reduces appetite. Changes in the digestive tract, including decreased acid production and decreased conversion of pepsinogen to pepsin, means that nutrients are extracted less efficiently from food. The breakdown of the tight junctions between epithelial cells causes a loss of metabolites into the gastrointestinal tract, especially protein, and finally protein is diverted to immune responses and tissue repair. The combination of the changes causes a relative protein deficiency [7]; this is particularly important because ruminants have evolved to feed on grass which is not a rich source of protein and the blood biochemistry of a healthy sheep is similar to that of a starved human being. Sheep fed a protein-rich diet (16% protein) show no clinical signs following infection with *Haemonchus contortus.* In contrast, sheep fed a standard diet (12% protein) and given the same number of *H. contortus* responded with weaker immune responses and developed severe clinical signs [46,47,48,49].

A variety of molecules including TSLP, IL-25 and IL-33 influence the Th2 response [36]. Epithelial cells are the major source of thymic stromal lymphopoietin (TSLP) [50,51] but fibroblasts, dendritic cells, basophils and mast cells also produce TSLP following stimulation. TSLP increases the expression of OX40L on dendritic cells [30]. IL-25 binds to IL-17Rβ which is expressed on CD4^+^ Th cells, ILC2 and dendritic cells. IL-25 acts together with IL-4 and STAT6 to promote the expression of GATA3. IL-33 is released from dead or damaged epithelial cells. It binds to ST2 on some activated Th2 cells and stimulates the production of type 2 cytokines. TSLP, IL-25 and IL-33 also activate ILC2. In addition, Th2 cells primed in draining lymph nodes require exposure to TSLP, IL-25 and IL-33 in the tissues to develop fully. IL-1β suppresses the production of IL-25 and inhibits the development of the Th2 response [52].

MicroRNA (miRNA) modulate translation by inhibiting and degrading mRNA molecules. Multiple miRNA influence gene expression in Th2 cells [30]. However, there is little overlap between in vitro generated Th2 cells and ex vivo Th2 cells from mice with house dust mite (HDM)-induced airway inflammation [53]. For example, miR-146a is down-regulated in in vitro generated Th2 cells but upregulated in Th2 cells ex vivo from mice with HDM induced airway inflammation or infected with the helminth *H. polygyrus.* Similarly, Th2 cells ex vivo from mice with HDM induced airway inflammation are distinct from Th2 cells from nematode infected mice [53].

The molecule miR-155 regulates several genes in Th2 cells and is required for Th2 mediated resistance to *H. polygyrus*, partly by downregulating the sphingosine-1-phosphate receptor 1 gene (*s1pr1*) [53]. Sphingosine-1-phosphate is a metabolite of plasma membrane glycolipids that plays an essential role in immune cell trafficking [54], especially in the migration of activated lymphocytes from lymph nodes. In contrast, miR-146a inhibits Th2 responses [53]. After infection with *Trichuris muris* or *H. polygyrus*, deletion of miR-146a in T cells led to a mixed Th1, Th2 and Th17 response and increased susceptibility to infection.

Other miRNA have also been implicated in the development of Th2 cells [30]. MiR-19a promotes Th2 development by suppressing negative regulators of TCR signalling (PTEN) and type 2 cytokine production (SOCS1). Two miRNA, miR-24 and miR-27, suppress Th2 development [55,56]. The former reduces expression of IL-4 by binding the 3′ UTR [56]; although this binding site is absent in humans. miR-27 represses *GATA3* [55,56]. Both miR-24 and miR-27 may also target additional molecules [55,56].

Macrophages play an important role in the response to nematode infection. Th1 responses produce classically activated macrophages (M1) while Th2 responses produce alternatively activated macrophages (M2) [57]. Other macrophage subpopulations also exist such as regulatory macrophages. However, precisely how M2 macrophages mediate protection against nematodes is unclear and may be related to their ability to dampen inflammation, promote wound healing and kill incoming larvae [57].

## 3. Protective Mechanisms Underlying Resistance to Nematode Infection

Resistant individuals can have fewer worms or smaller worms or both [40]. Individuals resistant to some nematodes may also have more inhibited larvae [58] but the number of inhibited larvae can also be influenced by the time of year, genetic variation in the parasite and by the size of the dose of infection [42,59]. It is now clear that different immune mechanisms underlie each component of resistance [40,60].

Th2 cells comprise several subpopulations but together they secrete cytokines including IL-4, IL-5 and IL-13. These cytokines drive the production of IgE, eosinophils and mast cells and possibly IgA, all of which play a critical role in protection against some nematodes [17]. There is considerable variation among hosts and among parasites in the mechanisms underlying resistance to infection. For example, in sheep, most of the variation in the size of adult *T. circumcincta* can be accounted for by variation in the intensity of the mucosal IgA and eosinophil response [60] while variation in the number of activated mast cells (globule leukocytes) is the most important source of variation in the number of worms following deliberate infection [60]. In contrast, *H. contortus* also occupies the abomasum of sheep but it feeds on blood. Consequently, antibodies in the blood can help protect against this parasite [61].

Other immune mechanisms may also contribute to resistance. Type 2 immune responses are also associated with increased mucus production and increased peristalsis in the intestine and both may play a role in resistance to some nematodes [62]. Another mechanism is increased epithelial cell turnover in the intestine [63].

The rate at which immune responses develop varies among species, among animals and among sites. For example, the ability to control the intestinal worm *T. colubriformis* develops more quickly than the ability to control the abomasal worm *H. contortus* [64]. Moreover, type 1 (immediate) hypersensitivity responses (IgE and mast cells) develop more slowly than the IgA and eosinophils responses against *T. circumcincta* [40,65].

Further research [66] has supported the idea that the ability of eosinophils to control worm fecundity is not restricted to sheep. In mice infected with *Nippostrongylus brasiliensis*, the IL-5 producing subclass of memory Th2 cells (CXCRC6^+^ST2^+^CD44^+^ Th2 cells) play a critical role and the mice express more major basic protein, a key molecule in eosinophil mediated immune attack.

## 4. Regulation of the Immune Response

Multiple mechanisms are used by hosts to regulate the immune response [17], including the reduction in antigen concentration as the infection is cleared, the release of IL-10 and transforming growth factor beta (TGFβ) by macrophages following the uptake of antigen-antibody complexes and the production of regulatory T cells. Regulatory T cells express a number of surface molecules that play key roles in regulating the immune response, including CD25, cytotoxic T-lymphocyte associated protein 4 (CTLA-4), inducible T-cell co-stimulator (ICOS) and T-cell immunoreceptor with Ig and ITIM domains (TIGIT) [67]. A variety of molecules assist the differentiation and proliferation of Treg including TGFβ and epidermal growth factor (EGF) [36].

Treg are a heterogeneous population [68]. Most Treg are CD4^+^ but some are CD8^+^ and these CD8^+^ Treg may play a critical role in susceptibility to some autoimmune diseases, such as multiple sclerosis [69]. Most Treg develop in the thymus and these Treg are called natural Treg. About 10% of peripheral CD4 T cells are natural Treg. Their main role seems to be ensuring tolerance of self-molecules [68]. Other Treg are induced from effector cells in the periphery and are sometimes called pTreg [68]. The main role of pTreg seems to be preventing excessive inflammation in tissues exposed to foreign molecules [68]. Consequently, the TCR repertoire is different for natural and induced Treg [68]. Th3 and Tr1 cells are subpopulations of induced Treg. Th3 are activated by TGF-β while Tr1 are activated by IL-10 [68]. The role of Th3 and Tr1 cells in nematode infection is unclear.

There is further heterogeneity among Treg in the effector T cells that they regulate. T-bet is a transcription factor expressed in Th1 cells and some Treg. The Treg that express T-bet express the inhibitory molecule TIGIT which binds CD155 to dendritic cells. This increases the production of IL-10. These Treg inhibit Th1 responses [68]. IRF-4 is a transcription factor expressed in Th2 cells and some Treg. It allows Treg to express ICOS and CTLA-4. These Treg inhibit Th2 responses. STAT3 is a transcription factor expressed in Th17 cells and some Treg. In Treg it promotes the expression of IL-10, Ebi3, granzyme and perforin-1. These Treg suppress Th17 responses [68].

The transcription factor FOXP3 defines Treg but the expression of this transcription factor is more stable in natural Treg compared to induced Treg [68]. The stability of FOXP3 expression is determined by methylation of the enhancer in the second intron of the FOXP3 gene [68].

Most natural Treg are schooled in the thymus but some can be induced outside the thymus including in the intestinal mucosa [36]. In the thymus, the binding of the newly formed TCR to self-peptides presented by MHC class II molecules is tested; cells expressing TCR with high affinity are deleted, cells expressing TCR with low affinity mature into helper T cells while cells expressing medium affinity TCR mature into Treg [36]. This system helps ensure that early in the response when antigen is relatively plentiful, the more numerous helper cells dominate but as antigen concentrations drop, Treg play an increasingly important role.

Treg use a variety of mechanisms to suppress immune responses [68]. Antigen-specific suppression is mainly due to the interaction of Treg and dendritic cells. The DC either becomes a tolerogenic cell or is unable to present specific antigen in the context of MHC class II molecules [68]. Mechanisms of antigen-specific suppression include the binding of CD80/CD86 on the DC surface by CTLA-4 which deprives potential responders of essential co-stimulatory signals. Treg increase indoleamine 2,3 dioxygenase (IDO) in the DC. This decreases tryptophan which is needed for the proliferation of T effector cells. Another mechanism underlying antigen-specific immunosuppression is the removal of specific antigen-class II MHC complexes from the DC surface [68].

Non-antigen-specific mechanisms include CD39 and CD73 on the Treg surface. These molecules convert ATP via ADP to AMP leading to an increased extracellular concentration of adenosine which is immunosuppressive [70]. Another non antigen-specific mechanism is the release by Treg of the immunosuppressive cytokines IL-10, IL-35 and TGF-β [68]. Treg can also disrupt the supply of calcium to effector lymphocytes; calcium is required for activation following TCR binding. Contact mechanisms include the release of perforin-granzyme by Treg to kill CD4 and CD8 T cells. Activated Treg express TNF-related apoptosis inducing ligand (TRAIL) which binds to death receptor 5 (DR5) on effector lymphocytes [68]. This binding activates caspase 8 in the effector cell and induces apoptosis. Treg express PD-1 which binds to PD-L1 on DC and makes them tolerogenic. Treg also express PD-L1 which binds to PD-1 on activated effector cells and causes anergy or their induction into pTreg. Moreover, Treg express a high affinity IL-2 receptor which soaks up the IL-2 needed by other T cells [36].

Treg are particularly relevant in some nematode infections [67]. Humans infected with a variety of nematodes have more natural Treg (defined as CD4^+^CD25^+^ cells [71] or CD4^+^CD25^+^FOXP3^+^ [72] or CD4^+^CD25^+^FOXP3^+^CD127^−^ [73]) compared to uninfected controls [67]. These include the filarial nematode *Wucheria bancrofti* [73], the hookworm *Necator americanus* [72] and the large intestinal worm *Ascaris lumbricoides* [71]. In mice, both *Litomosoides sigmodontis* [74] and *H. polygyrus* [75] infections are followed by an expansion of natural Treg. Depletion of Treg can enhance Th2 cytokine responses and parasite expulsion [74]. Depletion of regulatory T cells in low dose *T. muris* infection has a small but significant effect on parasite expulsion [76,77] although if Treg were depleted after parasites are established, the worm burden increased [76,77].

## 5. Immunomodulation

Nematodes produce a variety of molecules (Table 1) that modulate the host immune response [1,78]. Over 20 nematode-derived immunomodulators have been described [1]. Most of these have only been reported in one species of nematode although some such as apyrases [1,78], galectins [79] and cystatins [1] are more widespread among nematodes. However, these more widespread molecules have only been shown to be immunosuppressive in a small number of species. Only a small number of immunomodulators have been identified in each nematode, consistent with the idea that much of the immunomodulatory effect is due to a relatively small number of molecules.

These immunomodulators affect the immune response in many different ways, including hindering the induction of immune responses [80], inhibiting the expression of protective mechanisms such as mast cell degranulation [79,81] and promoting the induction of regulatory T cells [82]. In addition to effects mediated by specific parasite-derived proteins, lipids, RNA and small peptides also play a role [83]. An improved understanding of these molecules could lead to better methods of disease control (e.g., vaccination of humans and domestic animals, selective breeding of livestock) and may lead to immunomodulators that can treat autoimmune, inflammatory or allergic diseases.

Nematodes have been classified into five clades [84]. Immunomodulators have been described in several species of nematodes [1] from three clades. The immunomodulators will be discussed by family or superfamily within each clade to emphasise phylogenetic similarities and differences. The taxonomy follows that used at the EBI website (https://www.ebi.ac.uk (accessed on 3 January 2023)).

In clade I, the order Trichinellida contains the families Trichinellidae and Trichuridae, which contain the genera Trichinella and Trichuris, respectively. *Trichinella spiralis* secretes a mimic of macrophage migration inhibitory factor [85].

The secretions of Trichurid worms have been recently reviewed [63]. The excretory secretory fluid (E/S) of adult *T. muris* contains over 460 proteins [63]. The most abundant component of E/S is protein p43 which binds IL-13 and promotes parasite survival [63]. The pig whip worm *Trichuris suis* secretes Prostaglandin E2 (PGE2) and immunomodulates dendritic cells [86]. Two additional proteins that influence dendritic cell and macrophage secretion are triosephosphate isomerase and nucleoside diphophosphate kinase [87]. A chitinase from *T. suis* reduced clinical signs of airway disease in a mouse model, mainly by reducing eosinophil recruitment into the lung [88].

Extracellular vesicles (EV) include exosomes, microvesicles and apoptotic bodies. *T. muris* EV contain at least 70 proteins and 14 microRNA [63]. Bone-marrow-derived macrophages incubated with soluble product from homogenized adult *T. suis* (TSP) released more EV [89]. These EV contained more proteins than EV from control macrophages [90] and they reduced the production of TNF-α and IL-6 by macrophages [89]. Subsequent research by the same group showed that macrophages treated with Toll-like receptor (TLR) agonists and exposed to TSP also released IL-10 [90].

Some of the clade 3 nematodes cause lymphatic filariasis, one of the most serious parasitic diseases of humans. The three most important species in humans are *Brugia malayi*, *Brugia timori* and *Wucheria bancrofti*. Filarial nematodes belong to the superfamily Filaroidea and many pathogenic nematodes belong to the family Onchocercidae. This family includes the genera Brugia, Wuchereria, Onchocerca and Acanthocheilonema.

The filarial nematode *B. malayi* releases two molecules (Bm-MIF-1, Bm-MIF-2) with similar activity to human macrophage migration inhibitory factor although the amino acid (AA) sequences are only weakly similar (42% and 27% AA identity) [91,92]. They activate human monocytes and induce them to release IL-8, TNF-α and endogenous MIF [91]. Bm-MIF-1 contains 115 AA including the initial methionine and has a predicted molecular weight of 12,320. The accession number of Bm-MIF-1 (protein) is P91850 and it has one isoform (A0A1D5BKM6). The accession number of Bm-MIF-2 (nucleotide) is AY004865. The corresponding protein sequence has accession number Q9NAS2. There are four additional protein sequences for Bm-MIF-2; two of these are for a protein with 147 AA (A0A1U7FOUI and A0A4E9F914) and two for a protein with 120 AA (A0A1P6BWP3 and A0A4E9F776). The shared regions of A0A1U7FOUI, A0A4E9F914, A0A1P6BWP3 and A0A4E9F776 are identical but they differ from Q9NAS2 at position 79 where serine has been substituted for a leucine in Q9NAS2. Bm-MIF-1 (P91850) has only 27.8% AA identity with Bm-MIF-2 (Q9NAS2).

For MIF-1, the nematodes *B. timori*, *Brugia pahangi* contain identical sequences while *W. bancrofti* has a molecule that differs by only one AA at position 13 where Asparagine has been replaced by Aspartic acid. Other members of the family Onchocercidae with MIF-1 sequences include *Cercopithifilaria johnstoni*, *Acanthocheilonema viteae*, *Onchocerca flexuosa*, *Onchocerca ochengi*, *Onchocerca volvulus*, *L. sigmodontis* and *Loa loa*; the sequence with the lowest AA identity is *L. loa* at 86.1%. The most similar nematode from a different family is *Thelazia callipaeda* with 76.5% AA identity.

For MIF-2, *O. volvulus* contains a 120 AA sequence that is 76.7% identical to A0A1P6BWP3 while the 120 AA sequence in *T. callipaeda* has only 67.5% identity. MIF mimics have also been described in *Trichinella spiralis* and *Anisakis simplex* [1].

*B. malayi* also has a gene (*tgh-2*) which is a member of the TGF-β subfamily [93]. The C terminal domain shows 32% AA identity to human TGF-β1 and the recombinant protein binds to mink epithelial cells that expressed the TGF-β receptor [93]. This binding was partially inhibited by human TGF-β [93]. Two genes (*Bm-alt*-1 and *Bm-alt*-2; abundant larval transcript-1 and -2) account for 5% of the transcripts from *B. malayi* infective larvae [94]. These genes are similar with 79% sequence identity [94]. The two genes were cloned and separately expressed in free-living culture promastigotes of *Leishmania mexicana.* Transfection with abundant larval transcript (*alt*) genes improved the ability to infect macrophages in vitro [94]. In vivo, transfected parasites were more resistant to IFNγ-induced killing by macrophages and produced disease more quickly [94]. Infected macrophages produced more GATA-3 and SOCS-1 [94].

A subset of people infected with *B. malayi* develop elephantiasis. The transcription factor NF-κB regulates IL-8 which stimulates vascular endothelial growth factor (VEGF) [95]. VEGF contributes to lymphangiogenesis and the pathology [95].

*B. malayi* excrete asparaginyl t-RNA synthetase which blocks IL-8 receptors and increases IL-10 production [95]. This molecule is anti-inflammatory in a mouse model of colitis using T-cell transfer [95].

The KCNA3 protein provides a potassium voltage gated channel. During activation of T cells, it enables a potassium efflux that balances calcium signalling [96]. Bioinformatic analyses have identified a number of nematode-derived peptides and domains that resemble channel blockers [96]. Mimics were cloned from *B. malayi* and *Ancylostoma caninum.* The human parasite *Ancylostoma ceylanicum* has an identical 51 AA peptide to *A. caninum.* These peptides blocked the potassium channel and prevented proliferation of rat effector memory T cells [96]. They also inhibited the delayed-type hypersensitivity response due to the transfer of effector memory T cells [96].

Antigen presenting cells digest foreign molecules with multiple proteases including cysteine proteases. *B. malayi* contains a gene (*Bm-CPI-2*) that produces a cysteine protease inhibitor [97]. This cystatin molecule inhibited the presentation of T cell epitopes by antigen-presenting cells [97]. Cystatins are also produced by the nematodes *A. viteae*, *A. lumbricoides*, *O. volvulus*, *L. sigmodontis*, *H. polygyrus* and *N. brasiliensis* [1].

Microfilariae of *B. malayi* and *W. bancrofti* release prostaglandin E2 [98]. Subsequently, PGE2 was shown to induce OX40 ligand on DC [99] which allows dendritic cells to drive Th2 responses [99]. As discussed above, the pig whipworm *T. suis* also secretes PGE2 [86].

Other species within the superfamily Filaroidea have been shown to produce additional immunomodulators. The jird nematode *A. viteae* secretes a molecule with a molecular weight of 62,000 (ES-62). This phosphorylcholine containing molecule subverts TLR-4 signalling and down regulates MyD88 responses [100,101].

The most prevalent nematode infection of humans is due to *A. lumbricoides*; a member of the superfamily Ascarididae. In addition to the cystatins mentioned previously, a protein PAS-1 isolated from larval culture and adult body fluid has been shown to inhibit inflammation induced by Lipopolysaccharide in a mouse model by stimulating the production of IL-10 [102]. N-terminal sequencing identified an eleven AA peptide that was identical to a sequence of 11 AA in the ABA-1 polyprotein [102] previously described [103].

**Figure 1:**
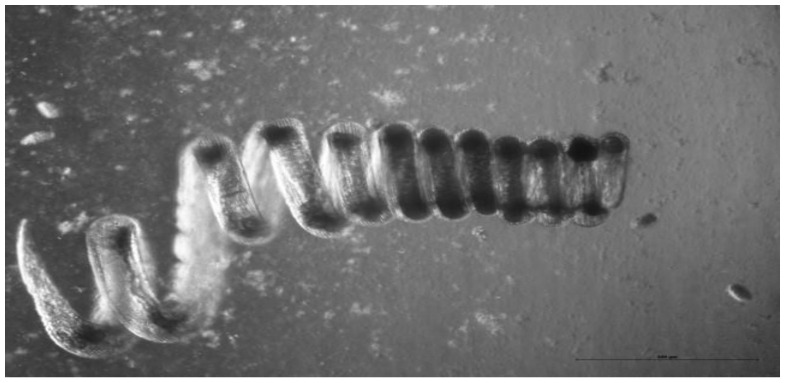
A female adult *H. polygyrus.* In vivo the worm wraps itself around intestinal villi which may help to prevent expulsion by peristalsis. Photo: K. Donskow-Lysoniewska.

The clade V nematodes include the superfamilies Ancylostomatoidea and Trichostrongyloidae. The mouse nematode *H. polygyrus* (Figure 1) is a member of the Trichostrongyloidea. It among the best understood of all nematode infections and immune modulation has been clearly demonstrated [37]. Vaccination with irradiated *H. polygyrus* larvae confers protection against subsequent challenge. However, coadministration of unirradiated larvae reduces the effectiveness of the irradiated larval vaccine. The simplest explanation is that live worms inhibit the expression of protective immunity [1].

*H. polygyrus* has a direct life-cycle [37]. Adults live in the small intestine where they breed; eggs are laid by adult females and excreted in the faeces. They develop through two moults into infective third-stage larvae. Infective larvae are ingested in natural infections but usually orally gavaged in experimental infections. Within 24 h, larvae have penetrated into the submucosa where they develop over the next ten days and undergo two moults before emerging into the lumen of the small intestine. Depending on the strain infected, about two weeks after infection, eggs can be seen in the faeces. The survival of adult parasites varies among mouse strains [37]. Some strains (SWR, SJL) expel parasites in 4–6 weeks, BALB/c mice expel parasites in 6–8 weeks while other strains (CBA, C3H) expel worms relatively slowly (greater than 20 weeks). Most strains of mice are resistant to re-infection but slow responder strains do not develop effective immunity against reinfection [37]. Immunity is mediated by the Th2 response [37]. Primary infection causes the expression of IL-3, IL-4 IL-5 and IL-9 in the mesenteric lymph nodes and Peyer’s patches [37]. Immunity to reinfection is reduced by antibody against IL-4 and abolished if the IL-4 receptor is also blocked [37], implying a role for IL-13 which also signals through the IL-4Rα molecule.

*H. polygyrus* produces at least five molecules that modulate immune responses [1] and these influence the immune response at several levels. The induction of the immune response is inhibited by a cysteine protease inhibitor that influences the differentiation of bone marrow derived dendritic cells [80], by an alarmin release inhibitor (HpARI) that prevents the release of the alarmin IL-33 from the nucleus where it is stored [104] and a miRNA that inhibits expression of the IL-33 receptor [105,106,107]. The expression of immunity is inhibited by galectin in some parasites [79] and our pilot trials show that this is also true for *H. polygyrus* (unpublished observations). Two molecules influence the production of regulatory T cells: TGM which is a TGFβ mimic [108], and a large immunosuppressive molecule (MW 226,476) recently discovered by us (EGF-M) that contains 24 Epidermal Growth Factor-like (EGF-like) domains, 2 SEA domains and one von Willebrand factor A domain. Our pilot trials suggest that this molecule or its cleavage products bind epidermal growth factor receptor (EGFR), disrupt normal signalling and modulate the immune response to nematode infection. The molecule also affects symptoms and clinical signs in autoimmune diseases (unpublished observations). We have called this molecule EGF-M (epidermal growth factor mimic).

*H. polygyrus* immunomodulators act on key stages of the immune response. Dendritic cells are essential for the development of the primary immune response [17] while IL-33 plays a key role in the induction of Th2 responses [109]. Mast cells play a key role in resistance to nematodes, including *H. polygyrus,* and both host and nematode galectins influence mast cell activity [79]. Both the EGF mimic and the TGFβ mimic can influence the differentiation and activation of regulatory T cells. EGFR and its ligands play important roles in resistance to nematode infection. Mast cell degranulation releases a serine protease that breaks the tight junctions between epithelial cells [110,111] and allows EGF to bind its receptors on the inner surface of the epithelium. This binding promotes repair processes including epithelial hyperplasia, reduced acid production, inappetence and plays a key role in the pathogenesis of gastrointestinal nematode infection [7]. In addition, Amphiregulin binds to EGFR, is essential for the production of the key cytokine IL-13 [112] and enhances resistance to nematode infection [113]. Amphiregulin also increases the activity of Treg [114]. Further, Heparin-binding epidermal growth factor also binds EGFR and influences the differentiation of T-cell subsets.

Another member of the Trichostrongyloidea is *H. contortus*, an economically important parasite of sheep, goats and cattle [115]. It develops in the abomasum and is a blood feeder [115]. It produces a molecule gp55 that inhibits neutrophils and monocytes [116]. This molecule is similar to a neutrophil inhibitory factor from *A. caninum* and *A. ceylanicum* [116,117] and cross-reacted with an antibody to the recombinant molecule from *A. caninum.* Another molecule from *H. contortus* with a molecular weight of 66kDa also inhibited monocyte function [118].

A third member of the Trichostrongyloidea is *T. circumcincta*, previously known as *Ostertagia circumcincta*. It produces a galectin that inhibits mast cell degranulation [81]. Galectins are the main extracellular molecules that bind glycans [79] and they influence the outcome of several different nematode infections [79,119]. Both vertebrates and invertebrates produce galectins but although structurally similar they do not appear to be descended from a common ancestor [120]. Host galectins play a key role in mast cell degranulation and possibly nematode galectins interfere with this process [81].

A fourth member of the Trichostrongyloidea is the rodent hookworm *N. brasiliensis* and this parasite is widely used as a model of human hookworm infections [57]. In addition to the previously mentioned cystatin, E/S fluid from adult *N. brasiliensis* digests platelet-activating factor [121], a multi-functional molecule that can enhance IgE mediated killing by eosinophils [121]. Many nematodes including *N. brasiliensis* secrete acetylcholinesterases [122]. Both B and T lymphocytes release acetylcholine and cholinergic signalling can affect the immune response [122]. Expression of acetylcholinesterase from *N. brasiliensis* in the natural mouse parasite *Trypanosoma musculi* changed the immune response [122]. Splenocytes from infected mice produced more IFN-γ and TNF-α but less IL-4, IL-5 and IL-13 [122]. There was classical activation of macrophages (M1) with enhanced nitric oxide production and decreased arginase activity [122]. Mice infected with transgenic *T. musculi* cleared infection more quickly [122].

Another superfamily in clade V is Ancylostomatoidea and this includes the dog hookworm *A. caninum*, the zoonotic hookworm *A. ceylanicum* and the human hookworms *Ancylostoma duodenale* and *N. americanus.* The potassium channel and the neutrophil inhibitory factor in Ancylostome species have already been mentioned. The gene encoding *A. ceylanicum* metalloprotease (*Ace-mtp2)* has been cloned [123]. The recombinant protein was incubated with an LPS-activated human leukaemia monocytic cell line; the protein induced derived macrophages to produce IFN-γ and increase TNF-α production [123].

Proteomic analysis of E/S from *A. caninum* identified two common proteins [124]: Tissue Inhibitor of Metalloprotease-1 and -2 (TIMP-1 and TIMP-2). These proteins are also known as Ac-AIP-1 and Ac-AIP-2 for *A. caninum* anti-inflammatory proteins. In vitro, recombinant AIP-1 suppressed the production of TNF-α and restored the production of IL-10 [124]. In vivo, it increased the number of regulatory cells in the colon and suppressed inflammation in a mouse model of colitis [124]. In mice, recombinant AIP-2 induced the expansion of CD103^+^ DC which generated Treg [125]. It also decreased the expression of MHC class II molecules on dendritic cells [125] In vivo, AIP-2 suppressed the infiltration of eosinophils and lymphocytes and reduced airway inflammation in a mouse model of asthma [125].

*N. americanus* produces one or more metalloproteinases that cleave eotaxin and are likely to interfere with eosinophil recruitment to the site of infection [126]. Another molecule Na-ASP-2 binds to CD79A, which with CD79B and immunoglobulin forms the B-cell antigen receptor [127]. This binding is followed by down-regulation of 1000 genes including three members of the B-cell receptor signalling pathway [127]. These findings suggest that Na-ASP-2 may interfere with antibody responses to infection with *N. americanus*.

Clearly, different species of nematodes use different strategies to modulate the immune response. This may be a consequence of the different effects of the immune response on each nematode species. To some extent, the differences may be exaggerated because few of the discovered immunomodulators have been tested in more than one species.

What is surprising and, so far, not explained, is the functional redundancy of the immunomodulators. Rather than developing multiple modulators that each influence distinct components of the immune response, evolution has often favoured the expression of sets of immunomodulators that act on the same stage of the immune response and are likely to influence each other. The interactions among immunomodulators have not been explored because most research has focussed on describing how single molecules modulate the immune response. Indeed, the potential interactions among the immunomodulators may be even greater than listed above. Regulatory T cells have receptors for IL-33 [109] and IL-33 induces proliferation of Treg and expression of amphiregulin [109]. Mast cells produce IL-33 [128] and release proteases that increase the activity of IL-33 [109]. Although much has been done, the study of parasite-derived immunomodulators is still in its infancy. The interactions between nematode derived immunomodulators have still to be investigated in detail. Currently, we lack a comprehensive understanding of the way nematodes modulate host immunity and enhance their survival. A broader understanding could facilitate the enhancement of protective immune responses against nematodes and could, with further research, lead to treatments that more effectively suppress pathogenic immune responses in autoimmune, inflammatory and allergic diseases.

## Figures and Tables

**Table 1:** Nematode-derived immunomodulators listed by species and by molecular or cellular target. References and further details are provided in the text.

Species	Immunomodulator	Target
*Trichinella spiralis*	Macrophage inhibitory factor mimic	Macrophages
*Trichuris muris*	p43	IL-13
*Trichuris suis*	Prostaglandin E2	Dendritic cells
*Trichuris suis*	Triosephosphate isomerase	Dendritic cells
*Trichuris suis*	Nucleoside diphophosphate kinase	Dendritic cells
*Trichuris suis*	Chitinase	Eosinophils
*Trichuris suis*	Extracellular vesicles	Macrophages
*Brugia malayi*	MIF-1; MIF-2	Macrophages
*Brugia malayi*	Tgh-2	TGFβ receptor
*Brugia malayi*	Alt-1; alt-2	Macrophages
*Brugia malayi*	asparaginyl t-RNA synthetase	Il-8 receptors; IL-10
*Brugia malayi; Wucheria bancrofti*	Prostaglandin E2	Dendritic cells
*Brugia malayi; Ancylostoma caninum*	KCNA3	T cells
*Brugia malayi; Acanthocheilonema viteae*; *Ascaris lumbricoides*; *Onchocerca volvulus*; *Litomosoides sigmodontis*; *Heligmosomoides polygyrus; Nippostrongylus brasiliensis*	Cystatin	Antigen presenting cells
*Acanthocheilonema viteae*	ES-62	TLR-4
*Ascaris lumbricoides*	PAS-1	IL-10
*Heligmosomoides polygyrus*	Alarmin release inhibitor	IL-33
*Heligmosomoides polygyrus*	mi-RNA	IL-33 receptor
*Heligmosomoides polygyrus*	TGM (a TGFβ mimic)	TGFβ receptor; Regulatory T cells
*Heligmosomoides polygyrus*	EGF-M (an EGF mimic)	EGF receptor; Regulatory T cells
*Heligmosomoides polygyrus; Teladorsagia circumcincta*	Galectin	Mast cells
*Haemonchus contortus*	Gp55; p66	Neutrophils and monocytes
*Haemonchus contortus*	p66	Monocytes
*Nippostrongylus brasiliensis*	Acetylcholinesterase	Acetylcholine
*Ancylostoma ceylanicum*	Metalloprotease-2 (mtp-2)	Macrophages
*Ancylostoma caninum*	Tissue inhibitor of metalloprotease -1,-2	Regulatory T cells
*Necator americanus*	Metalloprotease	Eotaxin
*Necator americanus*	ASP-2	B cell antigen receptor

## Data Availability

Not applicable.
